# Molecular Characterization of the glyA Gene From the Clinical Isolates of Tannerella forsythia

**DOI:** 10.7759/cureus.54909

**Published:** 2024-02-26

**Authors:** Sujitha A, A.S. Smiline Girija, Vijayashree J Priyadharsini

**Affiliations:** 1 Department of Microbiology, Saveetha Dental College and Hospitals, Saveetha Institute of Medical and Technical Sciences (SIMATS) Saveetha University, Chennai, IND; 2 Department of Clinical Genetics, Saveetha Dental College and Hospitals, Saveetha Institute of Medical and Technical Sciences (SIMATS) Saveetha University, Chennai, IND

**Keywords:** chronic periodontitis, oral health, glya, periodontitis, tannerella forsythia

## Abstract

Background: The *glyA* gene in *Tannerella forsythia *is attributed for its virulence by producing the enzyme serine hydroxymethyltransferase (SHMT), which plays a vital role in bacterial cell metabolism.

Objectives: The study is thus aimed to determine the frequency of the *glyA* gene from the clinical strains of *T. forsythia *isolated from periodontitis patients.

Materials and methods: Forty-five patients with varying degrees of periodontitis were included in the study, and the plaque samples collected from them were anaerobically processed by inoculating onto sterile anaerobic blood agar plates using a gaspak system, with incubation at 37°C for 5-7 days. The DNA was extracted from the obtained isolated colony, and PCR was performed to confirm the presence of the *glyA* gene.

Results: In total, 46.6% (*n *= 7) of the cases in group III aggressive periodontitis *(n = 15) *and 6.66% (*n = *1) in group II stage II periodontitis *(n = 15) *showed the presence of *T. forsythia*, and among them, 57.14% (*n *= 4) showed the presence of the *glyA* gene.

Conclusion: The findings of the study showed that the *glyA* gene may be associated with the pathogenesis of *T. forsythia* and could be thus a novel candidate for the future theragnostic approach to combat periodontitis.

## Introduction

Periodontal disease is the progressive degeneration of the periodontal soft and hard tissues, which is caused by the dysbiotic microbial communities and aberrant immune responses in the gingival and periodontal tissues [1]. Periodontitis starts as an irreversible damage of the gingiva leading to the chronic inflammatory disease state resulting in higher bacterial invasion. This evokes the host's defense mechanism against the periodontal pathogens and further inflammation due to the pro-inflammatory mediators. Finally, periodontitis causes the periodontium to lose its architecture, resulting in the loss of alveolar bone and the affected tooth [2]. Periodontitis is clinically graded into four stages: stage 1, initial (gingivitis); stage 2, moderate; stage 3, severe with potential for tooth loss (aggressive periodontitis); and stage 4, severe with potential for loss of all the teeth [3]. Only 10% of adult populations worldwide have the severe form of periodontal disease, which is more prevalent in the third and fourth decades of life, and approximately 50% of adult populations worldwide suffer from periodontal disease, primarily in its mild and moderate forms and with a prevalence ranging from 45% to 50% [4].

Various studies have previously been used to investigate the connection of potent organisms associated with the periodontal habitats. *Porphyromonas gingivalis, Aggregatibacter actinomycetemcomitans, Prevotella intermedia, Tannerella* *forsythia, *and* Fusobacterium nucleatum, *and* Treponema denticola* are few organisms that were frequently reported to be associated with the periodontal disease [5]. In particular, *P. gingivalis, T. forsythia, *and *T. denticola, *collectively known as the "red complex," are specifically linked to the severity of periodontal disorders [6]. Amidst these pathogens, a Gram-negative, filamentous, nonpigmenting, nonmotile, anaerobe *T. forsythia* has been linked to advanced forms of periodontal disease, such as severe and refractory periodontitis. Only a few of *T. forsythia*'s possible virulence factors have been identified so far such as *bspA*, surface (S-) layer, *mutS, lysS*, and *glyA*. As of now, the virulence factor known as bspA has been thoroughly studied and is known to mediate alveolar bone loss in mice [7]. Additionally, it causes infection co-aggregating with *F. nucleatum, *binding to fibronectin, interacting with extracellular matrix components and with epithelial cell attachment with further invasion [8]. The S-layer in *T. forsythia* has been shown to have multiple virulence effects, including co-aggregation with other oral bacteria and serum resistance [9]. However, not much studies have been documented on the *glyA *gene in *T. forsythia *in association with the periodontal disease. Experimental evidence prevails to show that *glyA* in other pathogens like *E. coli *encoding for serine hydroxymethyltransferase (SHMT), an enzyme that changes L-serine to glycine. Therefore, it is preferable to inactivate *glyA *in order to stop L-serine degradation when designing strains that produce L-serine. However, due to the resulting lack of glycine and C1 units, the majority of *glyA *knockout strains show slow cell growth, emphasizing its role in virulence [10].

The pathophysiology of *T. forsythia* in periodontitis is still not well understood due to the difficulty in its phenotypic characterization and genotypic variations [11]. Also, not much evidence-based studies prevail on the *glyA* gene in *T. forsythia. *This study is thus a first of its kind investigation that is aimed to detect the frequency of the *glyA *gene from the clinical strains of *T. forsythia* from the periodontitis patients showing various grades of the periodontal disease. 

## Materials and methods

Study population and sampling 

Forty-five patients (n) with three different stages of periodontitis such as group 1: gingivitis (n = 15); group II: stage II periodontitis (n = 15); and group III: aggressive periodontitis (n = 15), visiting the Department of Periodontics, Saveetha Dental College and Hospital were included in the study. A control group was not included as there were no studies evidencing the frequency of *T. forsythia *among the healthy individuals. Ethical clearance and informed consent were obtained prior to the start of the study. Demographic data on the age, gender, geographical location, clinical history, etc., were recorded before the collection of the samples. Plaque samples were collected in reproducible amounts and were immediately transferred to sterile Eppendorf tubes containing tryptic digest blood broth supplemented with 5% sheep blood, 5% vitamin K, and hemin. The samples were then sent straight to the microbiology laboratory for the anaerobic processing.

Isolation of *T. forsythia*

Plaque samples were plated onto anaerobic blood agar base supplemented with 5% sheep blood, 5% hemin, and 5% menadione, and the plates were placed in an anaerobic jar with a gaspak system. Incubation was done for five to seven days, at 37°C, and after incubation, the plates were checked for the presence of colonies with black pigmentation. Gram staining was performed to observe the morphology of the isolates.

Molecular detection of *T. forsythia *and the *glyA* gene

Using a QIAGEN extraction kit and the manufacturer's instructions, bacterial genomic DNA (50-100 µg/µl) was extracted from fresh *T. forsythia* cultures and was stored at -20°C. A 5.6 µl of double-distilled water and 7.8 ul of 2x Master Mix (Takara, Japan) were used to create 15 ul of the amplification reaction mixture. The PCR conditions were set for 36 cycles in a thermocycler (Eppendorf Mastercycler, Germany) with the annealing temperature set at 58°C. Specifically designed primers (0.1 µl-100 picomoles/µl) for both *T. forsythia* and *glyA *were added separately for confirmation (Table 1). The polymerase chain reaction (PCR) amplicons were confirmed using a 100-bp DNA ladder and were visualized in 1% agarose gel electrophoresis.

**Table 1:** Primer details to confirm Tannerella forsythia and glyA

Target gene	Primer sequence	Amplicon size (bp)
Tannerella forsythia	F: GGGTGAGTAACGCGTATGTAACCT R: GCCCATCCGCAACCAATAAA	127
glyA	F: TTCTCGGGACTGGGATTATG	189
	R: GCGTCGTTGAAGTGACGATA	

## Results

**Figure 1:**
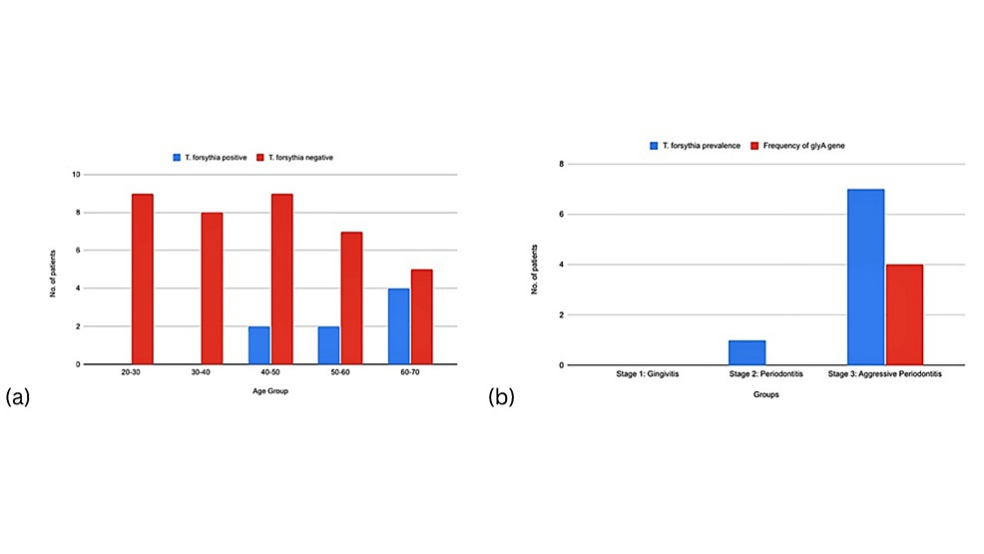
Prevalence of Tannerella forsythia in different age groups and gender

Patients in group III (n = 15) with aggressive periodontitis showed the maximum isolates (n = 7; 46.6%) followed by group II with one isolate (6.66%) of *T. forsythia *(Table 1).* *The frequency of the *glyA *gene was more in group III (57.14%) when compared to the other groups. Compared to younger patients, elder patients had a higher frequency of isolates. Plaque samples from patients in the 20-30 and 30-40 age groups did not show any growth of *T. forsythia. *However, the age group of 60-70 showed the highest number of isolates (n = 4), followed by two isolates in each of the 50-60 and 60-70 age groups (Figure 1).

**Figure 2:**
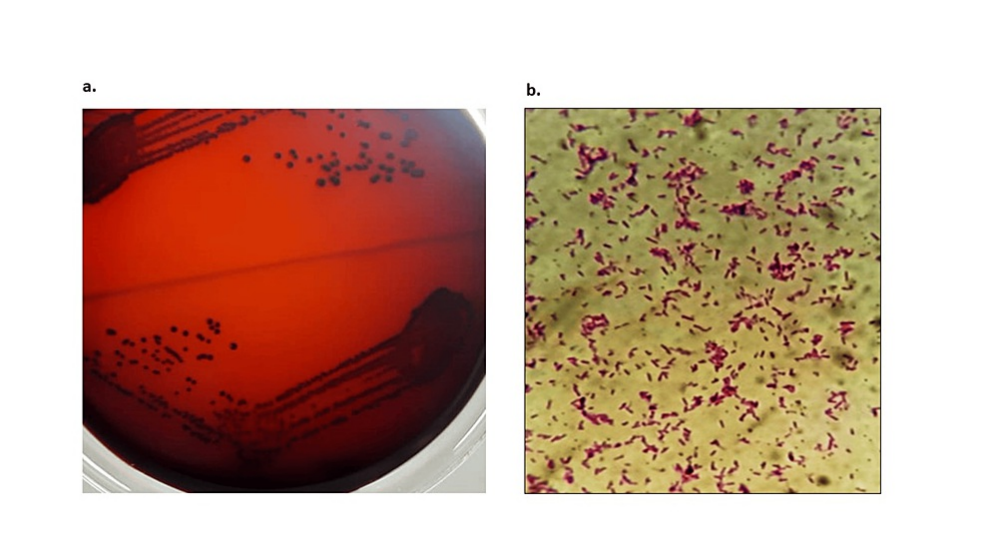
a. Typical black pigmented colony morphology of T. forsythia in anaerobic blood agar plate and b. Gram staining showing the pleomorphic Gram-negative bacilli

The colonies showed typical black pigmented colonies, and Gram staining showed Gram-negative pleomorphic bacilli (Figure 2).

**Figure 3:**
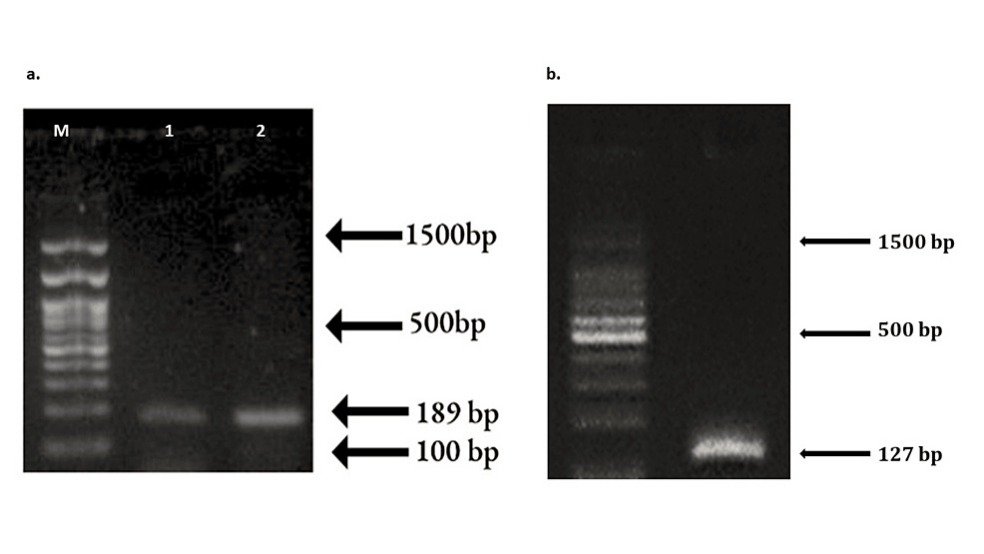
Agarose gel electrophoretogram showing (a) the amplification of glyA (amplicon size 189 bp) and (b) the genotypic confirmation of T. forsythia (amplicon size 127 bp), running along with standard DNA ladder (lane M = 100 bp DNA marker)

The isolates of *T. forsythia* and frequency of the glyA gene was further confirmed by molecular characterization using PCR (Figure 3).

Of the seven isolates in group III, the frequency of the *glyA *gene was observed as 57.14% (n = 4) with an amplicon size of 189 bp in group III and none of the other groups showed the presence of the gene.

## Discussion

Periodontitis is defined as a progressing and destructive disease of the periodontium, caused by a polymicrobial conglomeration of microbes influenced by the host factors. Patients with chronic periodontitis (CP) typically exhibit high levels of calculus and plaque, which correspond to the extent of periodontal damage. Aggressive periodontitis, on the other hand, is distinguished by a rapid rate of disease progression, with neither systemic involvement nor a familial accumulation of cases [12]. Rather than being a distinct condition, generalized aggressive periodontitis is most likely the previous stage of localized aggressive periodontitis [13]. Recent research has revealed the role of novel, noncultivable organisms in inducing the disease amidst functional biomes [14, 15]. One of the periodontal pathogens that has recently been linked to the etiology of periodontal diseases is *T. forsythia*. The pathogenesis and virulence of *T. forsythia* in colonizing the oral cavity are still unclear and yet to be elucidated.

In this context, it was recently documented that virulence factors like adhesions are associated with the cell surface of *T. forsythia* [16]. Animal studies have substantiated the same fact where *T. forsythia* resulted in alveolar bone loss in mice and rats [17] and skin abscesses in mice [18]. Additionally, there was a significant correlation of *T. forsythia* in aggressive periodontal cases from the subgingival plaque samples from the periodontitis-affected subjects [19]. In earlier studies, it was observed that subgingival plaque samples from the sites of periodontal disease showed higher rates of co-occurrence of *P. gingivalis*,* T. denticola*,and* T. forsythia* than from the samples from the healthy sites. The present study had also documented such similar results where the prevalence of *T. forsythia*was seen only in group II with aggressive periodontitis and not in the other two groups. In our study, we observed a higher correlation between the prevalence of *T. forsythia *in the aggressive periodontitis cases (46.6%) and a very less prevalence in group II (6.66%) with stage II periodontitis. These results substantiate the correlation of *T. forsythia* in association with disease severity. No strains were observed in group I patients with gingivitis portraying the less prevalence with increase in tissue involvement.

In an earlier study done in *E. coli*, SHMT, the product of the *glyA* gene, transforms serine into glycine [20] and is also reported to be responsible for lysostaphin resistance in *Staphylococcus aureus *[21]. However, no other studies have been evidenced to understand the role of *glyA *in *T. forsythia*. Thus, the present investigation is the first-of-its-kind study to reveal the frequency of this genetic determinant in association with different grades of *T. forsythia*. Prevalence of *glyA* was up to 57.14% (n = 4) from group III and was not found in the single strain isolated from group II patients. This finding also shows that *glyA* may be associated with the conditions of aggressive periodontitis and not indicated in the preliminary stages. With the prediction of putative vaccine candidates for priority pathogens being very common through computational approaches [22, 23], *glyA* may be considered as a novel target in the research arena of designing a periodontal vaccine [24]. The limitations of the study include the smaller sample size where we could not derive a significant result to substantiate our findings on the frequency of *glyA *in *T. forsythia* in association with periodontitis condition. Further experimental studies are to be designed in order to substantiate the correlation and association of *glyA* in *T. forsythia *with the periodontal disease.

## Conclusions

The findings of the present study thus give a clue on the presence of a higher frequency of *glyA *in *T. forsythia *in association with the aggressive periodontal disease condition. Periodic monitoring of such virulent determinants in all dental setups will aid in targeted therapy, diagnosis, and treatment of periodontitis. Frequent education programs for dentists creating awareness on the frequency of such virulent determinants would aid in curbing the complications of the disease as well as in the dental healthcare settings.
